# The Foreign Journals: Statistics

**Published:** 1840-10

**Authors:** 


### STATISTICS.

### *Longevity in Russia.*

In the government of Kasan there were, among the old people who died in 1839, 5 of 100, 7 of 101, 3 of 102, 3 of 103, 2 of 104, 3 of 105, 1 of 107, 2 of 108, 1 of 112, and 2 of 115 years old. In the government of Woronesch, there were 33 of 100, 11 of 105, 3 of 110, 3 of 115, and 2 of 120.

*Zeitschrift für die gesämmte Medicin. June,* 1840.

### *Mortality of St. Petersburg.*

An evident proof of the unhealthiness of the climate and mode of living of St. Petersburg is supplied by the extracts from the church books respecting the numbers born and dead in the foreign religious communities in the city, in the course of the year 1839. (The Greek church, Mahomedan, and Armenian congregations are excepted.)

| | Born. | | | Dead. | | |
|---|---|---|---|---|---|---|
| | Males. | Females. | Total. | Males. | Females. | Total. |
| St. Anne's Community | 148 | 128 | 276 | 130 | 124 | 254 |
| St. Catharine's | 155 | 131 | 286 | 158 | 133 | 291 |
| St. Peter's | 101 | 89 | 190 | 160 | 158 | 318 |
| Cadets | 50 | 41 | 191 | 55 | 48 | 103 |
| Engineer Cadet corps | 5 | 10 | 15 | 7 | 6 | 13 |
| Swedes | 61 | 60 | 121 | 108 | 101 | 209 |
| Finlanders* | 104 | 113 | 217 | 285 | 227 | 512 |
| English | 22 | 35 | 57 | 24 | 23 | 47 |
| German Reformed Church | 32 | 34 | 66 | 30 | 26 | 56 |
| French Reformed Church | 2 | 6 | 8 | 5 | 3 | 8 |
| Dutch | 2 | 5 | 7 | 3 | 3 | 6 |
| Estnische | 24 | 20 | 44 | 97 | 50 | 147 |
| Roman Catholics | 133 | 117 | 250 | 127 | 103 | 230 |
| Total | 839 | 789 | 1628 | 1189 | 1005 | 2194 |

*Zeitschrift für die gesämmte Medicin. June,* 1840.

* The Finlanders come in great numbers to be apprenticed at the various manufactories, and very frequently die of phthisis about the time of puberty.

### *Danish Medical Statistics.*

In the *Danish Medical Library* for January, February, and March, of the present year, there are copious extracts from the Transactions of the Royal Society of Health for 1839. The first is an account of the prevailing diseases in Denmark, with the exception of Laaland and Falster, during the year 1838. These are smallpox, scarlet fever, measles, catarrhal complaints, typhus, which has been very prevalent, rheumatic fever, puerperal fever, psora, syphilis, croup and hooping-cough, which last, after an absence of sixty years, has reappeared amongpe rsons of all ages in the Farœ Isles. We are also informed that in the same year (1838) 18,813 persons were vaccinated in the whole kingdom for the first time, and 1052 were revaccinated. That in Iceland 481 were vaccinated for the first time, and 647 were revaccinated.

The tables of births and deaths are not very precise. The number of accidental deaths and suicides, strangely enough united together, was 514.

*Bibliothek for Laeger. Jan. Feb. March,* 1840.

---

### II. THE BRITISH JOURNALS.

(FOR THE QUARTER ENDING AUGUST 31, 1840.)

---

